# Androgen receptor blockade using flutamide skewed sex ratio of litters in mice

**Published:** 2016-06-15

**Authors:** Faramarz Gharagozlou, Reza Youssefi, Mehdi Vojgani, Vahid Akbarinejad, Ghazaleh Rafiee

**Affiliations:** 1 *Department of Theriogenology, Faculty of Veterinary Medicine, University of Tehran, Tehran, Iran; *; 2 *Young Researchers and Elites Club, Roudehen Branch, Islamic Azad University, Roudehen, Iran.*

**Keywords:** Androgen receptor, Flutamide, Mice, Sex ratio

## Abstract

Maternal testosterone has been indicated to affect sex ratio of offspring. The present study was conducted to elucidate the role of androgen receptor in this regard by blockade of androgen receptor using flutamide in female mice. Mice were randomly assigned to two experimental groups. Mice in the control (n = 20) and treatment (n = 20) groups received 8 IU equine chorionic gonadotropin (eCG) followed by human chorionic gonadotropin (hCG) injection (8 IU) 47 hr later. In addition, mice in the control and treatment groups received four injections of ethanol-saline vehicle and flutamide solution (2.50 mg), respectively, started from 1 hr before eCG injection until hCG injection at 12-hr intervals. Conception rate was not different between the treatment (18/20: 90.00%) and control (19/20: 95.00%) groups (*p* > 0.05). Litter size was higher in the treatment (8.22 ± 0.26) than control (7.21 ± 0.28) group (*p* < 0.05). Male sex ratio was lower in the flutamide-treated mice (67/148: 45.30%) as compared with the untreated ones (80/137: 58.40%; odds ratio = 1.69; *p* < 0.05). In conclusion, the results showed that androgen receptor blockade could skew sex ratio of offspring toward females implying that the effect of testosterone on sex ratio might be through binding to androgen receptor. In addition, the blockade of androgen receptor using flutamide appeared to enhance litter size.

## Introduction

Numerous studies have indicated skewness in the expected equal proportion of sexes (50:50) at birth in different mammals,^1^^,^^2^ and this phenomenon has been attributed to various factors including maternal body condition,^3^ nutrition,^1^ maternal hormonal profile,^2^^,^^4^^,^^5^ maternal glucose concentration^4^^,^^6^ and stress.^7^^,^^8^

One of the main factors indicated to affect the sex is maternal dominance, i.e. more dominant mothers are more likely to produce males than females.^9^ Maternal dominance has been associated with maternal testosterone concentration.^10^^,^^11^ In this context, association between maternal testosterone and producing male gender has been reported in the vole,^4^ ibex^5^ and bovine.^12^^,^^13^ Yet it has remained unknown whether the effect of testosterone on the sex of offspring is through its direct effect on oocyte or through its conversion to a secondary molecule which would subsequently affect the sex of offspring. For example, treatment with oestradiol, which results from aromatization of testosterone,^14^ has resulted in male-biased sex ratio in mice^15^ and bovine.^16^

It has been indicated that it is pre-conceptional testosterone associated mechanisms rendering the oocytes to be more likely to be fertilized by Y than X chromosome-bearing spermatozoa.^12^^,^^13^ Therefore, the present study was conducted to evaluate the effect of pre-ovulatory administration of flutamide, an androgen receptor antagonist,^17^^,^^18^ on sex ratio of litters in mice (the proportion of males) in order to elucidate whether the action of testosterone on the gender is through binding to androgen receptor.

## Materials and Methods

**Animals and experimental design.** The mice (n = 40; aged approximately 12 weeks) were maintained in a temperature-controlled environment under 12 hr light/ 12 hr darkness photoperiod, and had *ad libitum* access to food and water. This study was approved by Faculty of Veterinary Medicine, University of Tehran, Tehran, Iran to secure its compliance with the Guide for the Care and Use of Laboratory Animals.^19^

The mice were randomly assigned to two experimental groups. The mice in the control (n = 20) and treatment (n = 20) groups received a subcutaneous injection of 8 IU equine chorionic gonadotropin (eCG; Hipra, Amer, Spain) followed by an intraperitoneal injection of 8 IU human chorionic gonadotropin (hCG; IBSA, Lugano, Switzerland) 47 hr later. Additionally, mice in the control and treatment groups received four subcutaneous injections of ethanol-saline vehicle and flutamide solution (2.50 mg; Iran Hormone Pharmaceutical Co., Tehran, Iran), respectively, beginning from 1 hr prior to eCG injection until hCG injection at 12 hr intervals. Flutamide solution was prepared by dissolving flutamide in 95.00% ethanol at 50.00 mg mL^-1^ concentration, which was further diluted with an equal volume of saline. Then, female mice were introduced to males; one male was caged with two female mice of different experimental groups. The sex of litters was determined by evaluating anogenital distance two days after birth, which was further confirmed at weaning.^20^

**Reproductive parameters.** Conception rate was defined as the number of mice conceived divided by the number of mice assigned to the study. Sex ratio was defined as the number of male litters divided by the number of all litters born.

**Statistical analysis.** Data associated with the binary outcome variables including conception rate and sex ratio were analyzed by logistic regression analysis using GENMOD procedure including function link logit in the model. Logistic regression analyses generated odds ratios as the estimates of strength of difference. Data associated with litter size was analyzed using TTEST procedure. All analyses were conducted in SAS (version 9.2; SAS Institute Inc.; Cary, USA). Differences were considered statistically significant at *p* < 0.05 level.

## Results

Out of 20 mice assigned into each group, 19 and 18 mice conceived in the control and treatment groups, respectively; there was no difference between two groups in conception rate (*p* > 0.05). Litter size was higher in the flutamide-treated (8.22 ± 0.26) than untreated (7.21 ± 0.28) mice (*p* < 0.05). Male sex ratio was lower in the treatment group (67/148: 45.30%) as compared with the control group (80/137 = 58.40%; odds ratio = 1.69; *p* < 0.05; Table 1).

**Table 1:** Parameter estimates, standard errors (SE), estimated odds ratios (OR) and 95% confidence intervals (CI) for the effect of flutamide on sex ratio of litters and conception rate in mice

**Item**	**Class **	**Parameter (%)**	**Estimate ± SE**	**OR**	**95% CI**	***p*** **-value**
**Sex ratio**	Ethanol-saline vehicle	80/137 (58.40)	0.52 ± 0.23	1.69	1.06 - 2.71	0.02
	Flutamide	67/148 (45.30)	―	Reference	―	―
**Conception rate**	Ethanol-saline vehicle	19/20 (95.00)	0.74 ± 1.26	2.11	0.17 - 25.33	0.55
	Flutamide	18/20 (90.00)	―	Reference	―	―

## Discussion

Female voles with high concentrations of circulatory testosterone have been observed to produce male-biased sex ratio.^4^ In addition, Shargal *et al*. found an association between fecal testosterone concentration and the sex of offspring being male in ibex.^5^ The association between testosterone and probability of the *in vitro* produced male embryos has been observed in bovine. Moreover, it has been shown that maternal testosterone impacts the sex ratio of offspring pre-conceptionally as oocytes originated from the follicles with high intrafollicular testosterone were more likely to produce male than female embryos.^12^^,^^13^ The present study revealed that this effect of testosterone on sex ratio of offspring might be mediated through androgen receptor, as pre-ovulatory treatment of mice with flutamide, which is an androgen receptor antagonist,^17^^,^^18^ resulted in a female-biased sex ratio. The binding of testosterone to androgen receptor results in dimerization of the receptor leading to binding of the androgen receptor to its cognate response element and recruiting co-regulators to promote the expression of target genes.^21^ Nevertheless, the downstream mechanisms ensuing binding of testosterone to androgen receptor leading to increase in the probability of fertilizing oocytes by Y chromosome-bearing spermatozoa over X chromosome-bearing ones are unknown and require further molecular studies to be elucidated.

Moreover, treatment with flutamide increased litter size in mice in the present study. Increasing ovarian androgen secretion, eCG has been observed to reduce oocyte quality, which has been indicated to be eliminated by flutamide.^17^ Therefore, higher litter size in flutamide-treated than untreated mice in the present study could be attributed to anti-androgenic effects of flutamide, enhancing oocyte quality.

Flutamide has been observed to interfere with LH surge, thereby blocking ovulation in rats.^18^ The demolishing effect of flutamide on ovulation was found to be abolished by hCG injection,^18^ which was probably the reason conception rates were not different between flutamide-treated and untreated mice in the present study.

In conclusion, the present study revealed that blockade of androgen receptor using flutamide could skew sex ratio of offspring toward females which implicates that the effect of testosterone on sex ratio is, at least in part, through binding to androgen receptor. Moreover, flutamide treatment improved litter size in mice.
